# Supplementary material for: Exploration of the ocular surface infection by SARS-CoV-2 and implications for corneal donation: An ex vivo study
Source: PLoS Med. 2022 Mar 1;19(3):e1003922. doi: 10.1371/journal.pmed.1003922 (PMC8887728; doi:10.1371/journal.pmed.1003922)
Supplement: S1 File — (A) Immunohistochemistry protocols validation. (B) Ex vivo experimental infection of fresh corneas with SARS-CoV-2. (C) SARS-CoV-2 RNA detection in the ocular surface tissues from COVID-19 and non-COVID-19 donors. COVID-19, Coronavirus Disease 2019; SARS-CoV-2, Severe Acute Respiratory Syndrome Coronavirus 2. (DOCX) [file pmed.1003922.s002.docx]

**Supporting information**

**Section A - Immunohistochemistry protocols validation**

*Methods*

The preliminary screening of the best antibodies was performed on VERO E6 cells. This cell type, known to express the markers of interest[**1**], allowed us to avoid false negative results. Indeed, the expression of the 4 cellular markers of interest at the ocular surface or inter-individual variability in expression was still not fully known and the use of corneal samples could be misleading. Additionally, a co-labelling with validated cell markers was performed to control and validate the experimental conditions. Finally, to limit erroneous positive observations, negative control conditions using mouse and/or rabbit IgG in replacement of the tested antibodies were evaluated on the tested cells and ocular tissues.

Cells were fixed at room temperature during 15 min and two conditions of fixation were tested: 4% paraformaldehyde (PFA) and pure methanol. Conjunctival epithelial cells collected by impression cytology (n = 8 samples) were further used to evaluate the selected antibodies with the relevant validated fixation protocol for each antibody (PFA or methanol). After permeabilization in 0.5% X-100 Triton, cells were sequentially incubated during 1h at 37°C with primary then secondary antibody.

The labelling of the four receptor/activators of SARS-CoV-2 was further explored on flat mounted non-infected human corneas as previously described[**2**]. This technique, complementary to classical immunostaining on tissue sections, provided not only a global mapping and a more precise localization of cells but also the visualization of rare cell types like immune cells at the ocular surface. Corneas were fixed in methanol or 0.5% PFA during 1h at room temperature, then permeabilized in 0.5% x-100 Triton. Fixed tissues were then incubated overnight at 4°C (1/200 dilution) with primary antibody while secondary antibody incubation was performed at 37°C during 2h (1/500 dilution). In a few samples, ACE-2 and TMPRSS2 were co-labeled with CD45 receptors (M0855, Dako, les Ulis, France).

*Results*

The labelling of ACE-2, TMPRSS2 and cathepsin L was well detected in Vero E6 cells whether fixed with PFA or methanol, whereas cathepsin B was only visible in PFA fixed cells (S1A Fig). Similarly, Vero E6 infected cells presented visible spike protein labelling in both fixation conditions whereas capsid protein labelling was detected only in methanol fixed cells (S1B Fig). Concerning the subcellular localization of the markers, ACE-2 labelling was homogenous and distinctly visible at the plasma membrane, whereas the labelling of cathepsin B at the cell membrane was punctiform. TMPRSS2 showed both membrane and cytoplasmic labelling whereas cathepsin L labelling was mostly localized in cytoplasm. The precise localization of the target proteins labelling was further explored on impression cytology samples and orthogonal view confirmed a clear and homogenous cell membrane labelling for TMPRSS2 (S2A3 Fig) and ACE-2 (S2B Fig). A distinct punctiform labelling of cathepsin B was localized at the cell membrane whereas cathepsin L labelling was observed in the cytoplasm (S2B Fig).

In these impression cytology samples, a strong staining of TMPRSS2 was observed in conjunctival epithelial cells, with a heterogeneous signal among cells. Co-staining of CK12 and TMPRSS2 was sparse (S2A1-2 Fig). Subcellular localization of markers was visualized on confocal microscopy orthogonal views: TMPRSS2 (S2A3 Fig) and ACE-2 localized at the cytoplasmic membrane, while cathepsin B and L were found in the cytoplasm (S2B Fig). A low inter-individual variability was noted for ACE-2 (11/11 samples), TMPRSS2 (10/11) and cathepsin L (11/11), while cathepsin B was more variably expressed (7/11). The higher positivity ratio using flat mounted impression cytology compared to immunohistochemistry on corneal cross sections could be explained by the much higher number of superficial cells visible on flat mounts than on cross sections allowing visualization of heterogeneous expression.

**Section B - Ex vivo experimental infection of fresh corneas with SARS-CoV-2**

*RT-PCR detection for SARS-CoV-2 RNA*

Fold changes in IP4 total number of copies between H0 and H24 for each donor were represented in S4 Fig.

*Transmission and scanning electron microscopy*

In order to detect Sars-Cov-2 particles at the ocular surface, both transmission (TEM) and scanning electron microscopy (SEM) were performed on central corneas and corneoscleral rims from *ex vivo* infection (after 30min of infection (H0) or on not infected tissues). Fresh tissues were used to explore viral fixation and cellular internalization whereas fixation of tissues was used to inhibit the process of viral internalization.

Just after the end of incubation with the viral solution, samples were washed 5 times in PBS before fixation. After fixation by 1% glutaraldehyde /0.5% PFA in 0.1 M Na/diK mono buffer (pH 7.4), samples were post-fixed in 1% osmium tetroxide (in 0.1M cacodylate buffer) during 1h, dehydrated in ethanol, and subsequently embedded in epoxy resin for TEM. Ultrathin sections (90 nm thick) were contrasted with uranyl acetate/lead citrate before analysis on a transmission electron microscope (H-800; Hitachi, Tokyo, Japon) equipped with a CCD camera (XR40, AMT, Danvers, Massachusetts, USA).

For SEM, samples were fixed in 0.1 N glutaraldehyde 2% (in 0.2N cacodylate buffer, pH 7.4) overnight at 4°C, rinsed with 0.2N cacodylate buffer then distilled water. After fixation, the samples were dehydrated progressively in different percentages of ethanol. The samples were immersed for 10 min in 100% hexamethyldisilazan after the 100% ethanol step. After air drying, samples were coated with Gold/Palladium by sputtering (Polaron SC 7620, Quorum Technologies) and analyzed on a scanning electron microscope (Hitachi S-3000N).

Representative SEM and TEM images of infected and non-infected corneal limbus were presented in S6 Fig. TEM images showed numerous structures comparable to previously described microplicae and microvilli at the surface of epithelial cells[**3**], with dimensions ranging from 50 to 200 nm. Due to their morphology and size very similar to viral particles, these structures could be easily mistaken for viral particles. On SEM Images, nanofilaments-like structures were observed at the surface of epithelial cells and were very similar to SARS-CoV-2 particles. Taken together, these observations do not permit concluding on the presence of SARS-CoV-2 particle in the studied samples.

*Immunostaining of viral protein in ex vivo infected corneas*

In parallel to electron microscopy, viral particle detection was tested by immunostaining using antibodies against proteins Spike 3525 and Capside 40143 previously validated on Vero E6 cells infected by SARS-CoV-2 (S1 Fig). Immunostaining of viral proteins was thus performed on *ex vivo* infected limbal epithelium after 24 hours of incubation with SARS-CoV-2 (S7 Fig). Only small areas of conjunctival epithelium were labeled for viral nucleocapsid. As a non-specific labelling was observed in non-infected tissues and only low labelling of viral particles was observable, current immunostaining methodology and conditions did not allow concluding on the presence of SARS-CoV-2 in *ex vivo* infected corneas. Concerning the immunostaining of viral Spike protein, a non-specific low labelling was observed in both infected and control non-infected samples, which did not allow us concluding on the virus presence. Thus, viral proteins immunostaining was abandoned.

**Section C - SARS-CoV-2 RNA detection in the ocular surface tissues from COVID-19 and non-COVID-19 donors**

Consistency between the two extraction kits was experimentally validated by using both kits in the same panel of samples, followed by quantification by RT-qPCR. The panel consisted in serial dilutions of whole-genome SARS-CoV-2 RNA sequence (Eurogentec) from 10^10^ to 10^4^ strand/ml, two positive controls consisting in the culture medium of two ex-vivo infected scleral samples, two negative controls consisting in portion of two corneas collected in two non-affected patients (SARS-CoV-2 PCR negative on nasopharyngeal swabs). In addition, classical negative controls (water replacing the sample and water without reaction mix) were also analyzed. Total RNA from each sample was extracted using both kits following the respective manufacturer’s instruction. After RNA extraction, the same protocol was followed for all samples, as detailed in the main Method section of the article. Ct values obtained for a same sample extracted with the 2 kits differed by a maximum 2 units (table S6).

**References:**

1. Zecha J, Lee CY, Bayer FP, Meng C, Grass V, Zerweck J, et al. Data, Reagents, Assays and Merits of Proteomics for SARS-CoV-2 Research and Testing. Mol Cell Proteomics. 2020;19(9):1503-22. Epub 2020/06/28. doi: 10.1074/mcp.RA120.002164. PubMed PMID: 32591346; PubMed Central PMCID: PMCPMC7780043.

2. He Z, Campolmi N, Ha Thi BM, Dumollard JM, Peoc'h M, Garraud O, et al. Optimization of immunolocalization of cell cycle proteins in human corneal endothelial cells. Mol Vis. 2011;17:3494-511. Epub 2012/01/06. PubMed PMID: 22219645; PubMed Central PMCID: PMCPMC3249439.

3. Pfister RR, Burstein NL. The normal and abnormal human corneal epithelial surface: a scanning electron microscope study. Invest Ophthalmol Vis Sci. 1977;16(7):614-22. Epub 1977/07/01. PubMed PMID: 873722.
