# Supplementary figures and images for: Exploration of the ocular surface infection by SARS-CoV-2 and implications for corneal donation: An ex vivo study
Source: PLoS Med. 2022 Mar 1;19(3):e1003922. doi: 10.1371/journal.pmed.1003922 (PMC8887728; doi:10.1371/journal.pmed.1003922)

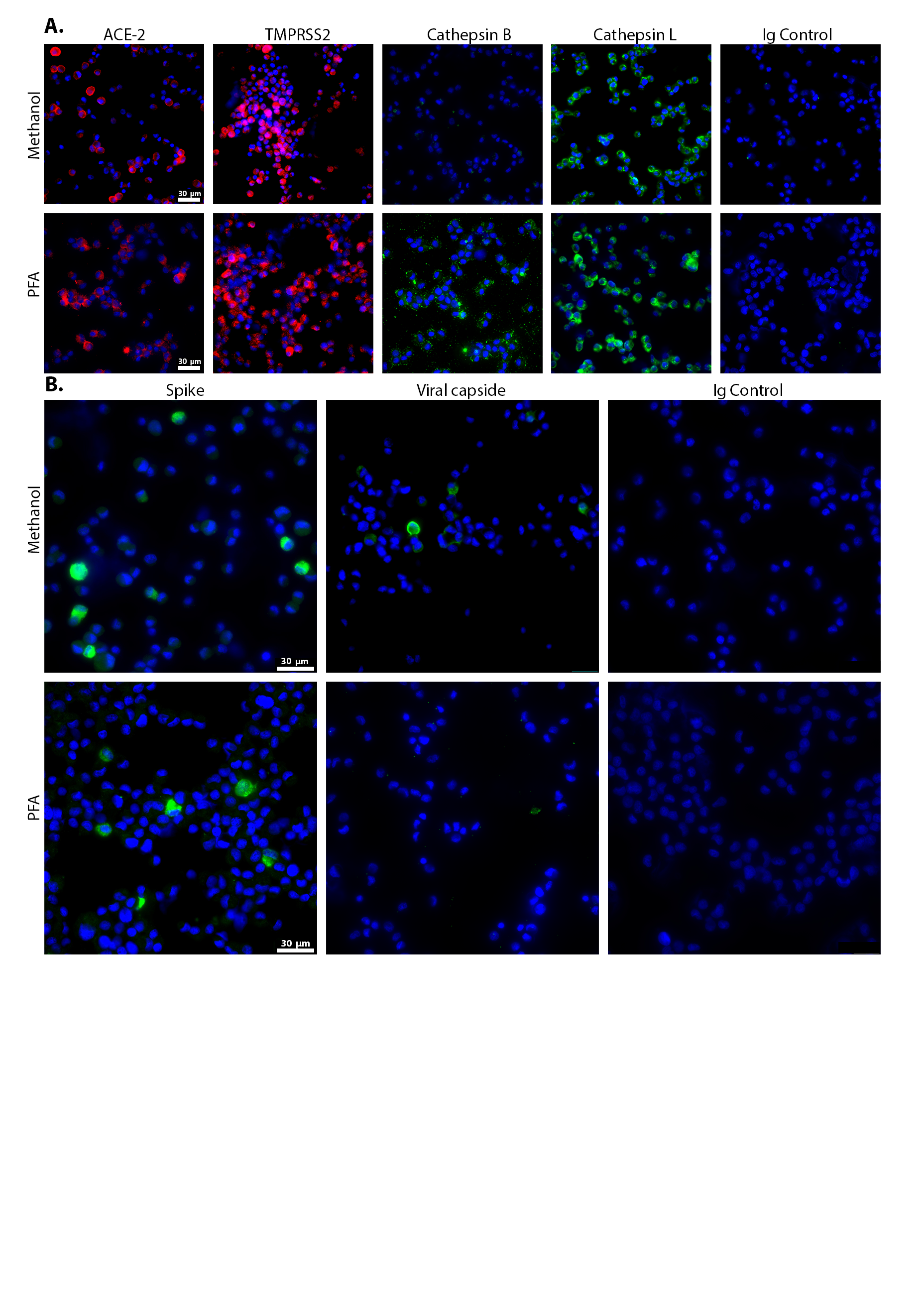

Supplement: S1 Fig — Representative immunostaining results obtained with the selected panel of primary antibodies targeting ACE-2, TMPRSS2, Cathepsin B, and Cathepsin L in VERO E6 cells (A) and targeting viral proteins Spike 3525 and Capside 40143 on VERO E6 cells infected by a clinical strain of SARS-CoV-2 (B). Immunostaining was tested on both methanol and PFA-fixed cells. ACE-2, angiotensin-converting enzyme 2; PFA, paraformaldehyde; SARS-CoV-2, Severe Acute Respiratory Syndrome Coronavirus 2. (TIF) [file pmed.1003922.s003.tif]

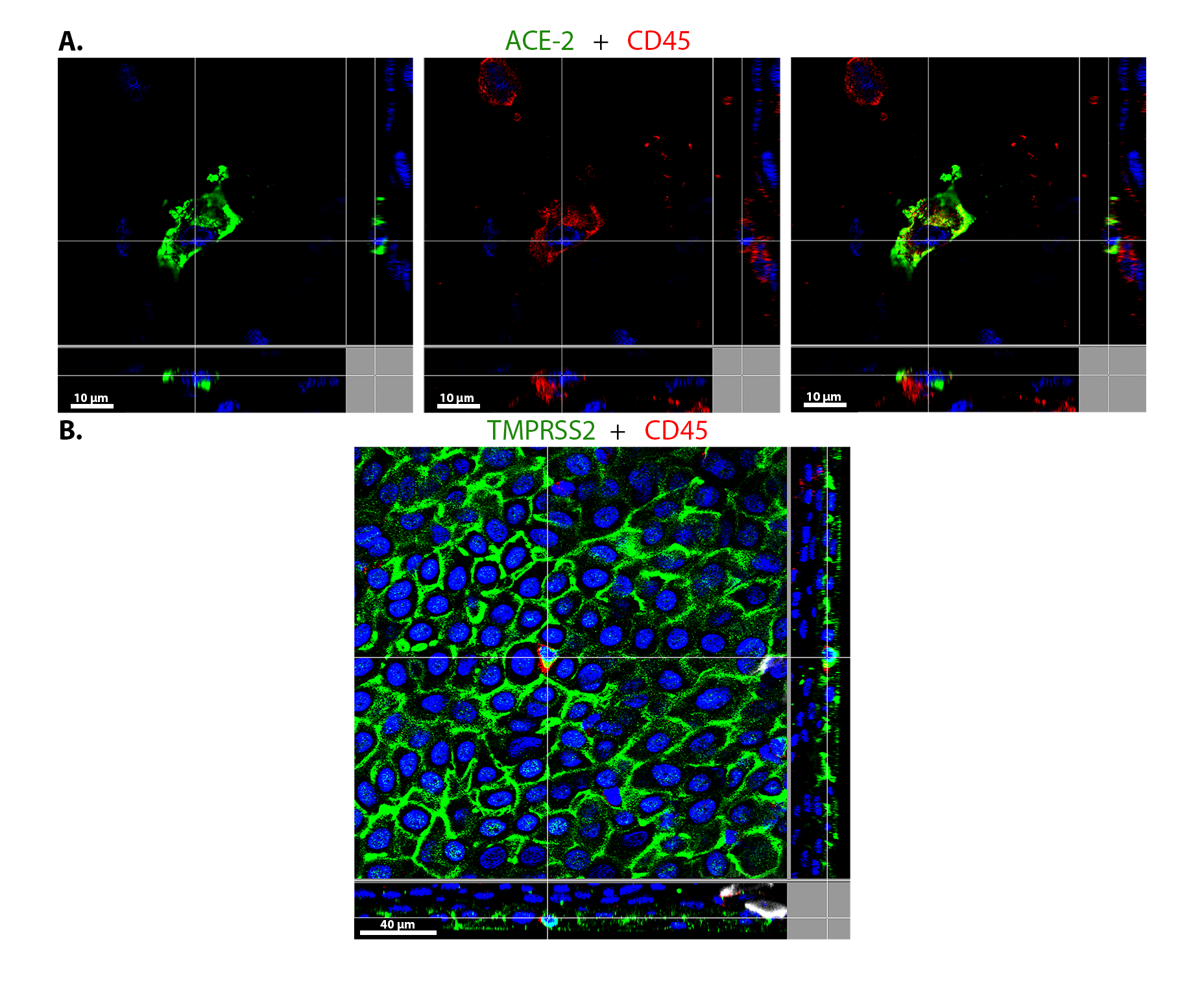

Supplement: S3 Fig — Immunostaining of ACE-2 or TMPRSS2 (green, Alexa 488) and CD45 receptor (red, Alexa 555) in the corneal epithelium (A) and conjunctival epithelium (B) from a flat mounted cornea of a healthy donor. ACE-2, angiotensin-converting enzyme 2. (TIF) [file pmed.1003922.s005.tif]

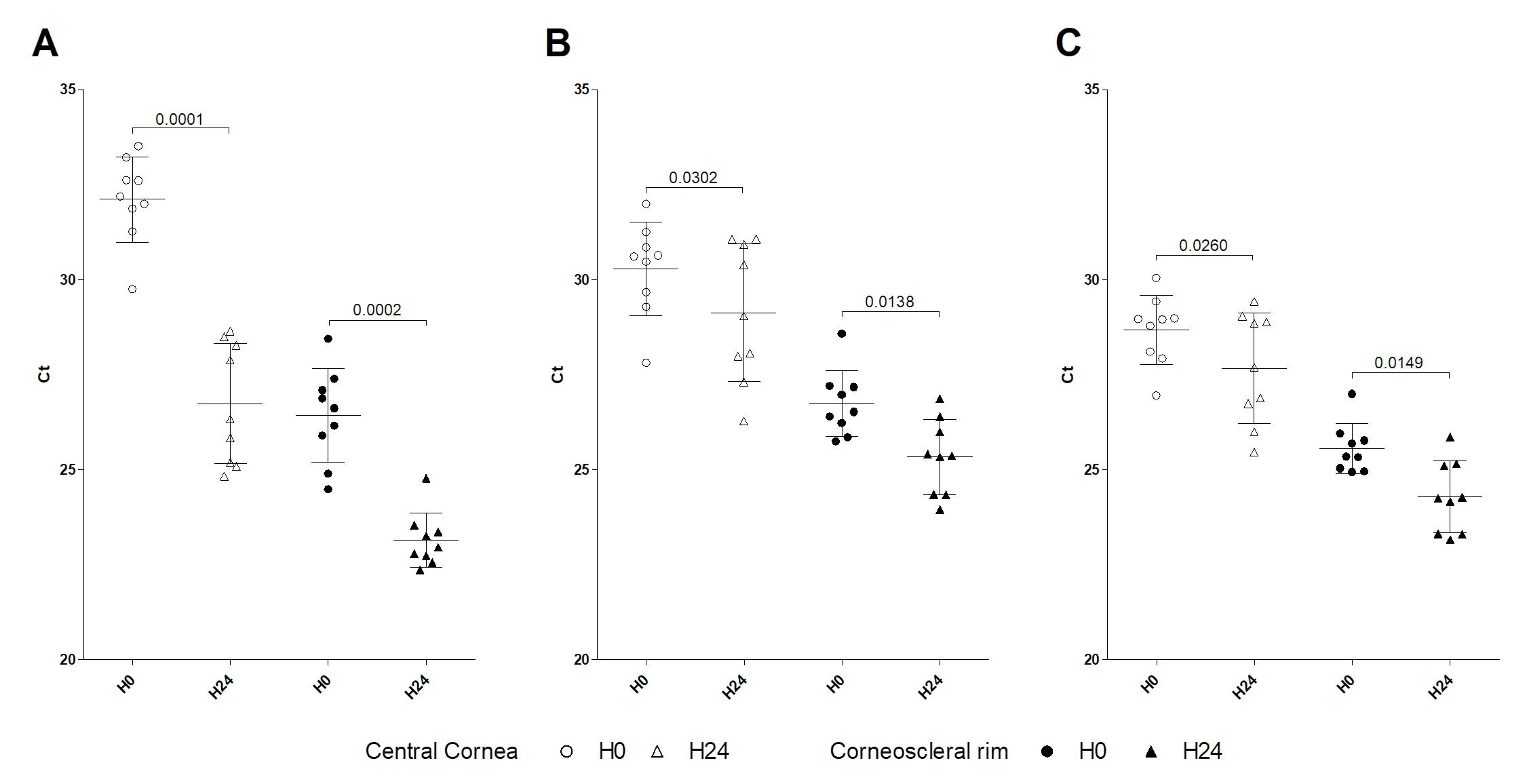

Supplement: S5 Fig — SARS-CoV-2 RNA expression as mean Ct ± SD of Cov-IP4-RdRp-2020 PCR (A), of Cov-N-2021 PCR (B) and of Cov-RdRp-2021 PCR (C) in the incubation media of central cornea and corneoscleral rim at 30 minutes (H0) and 24 hours (H24) after SARS-CoV-2 ex vivo infection. Results are provided for the respective culture medium of central cornea and corneoscleral rim. H0 and H24 data were compared using a paired t test. Ct, cycle threshold; SARS-CoV-2, Severe Acute Respiratory Syndrome Coronavirus 2. (TIF) [file pmed.1003922.s007.tif]
