# Supplementary material for: Exploration of the ocular surface infection by SARS-CoV-2 and implications for corneal donation: An ex vivo study
Source: PLoS Med. 2022 Mar 1;19(3):e1003922. doi: 10.1371/journal.pmed.1003922 (PMC8887728; doi:10.1371/journal.pmed.1003922)
Supplement: S1 Table — (DOCX) [file pmed.1003922.s010.docx]

**S1 Table**. List of primary antibodies validated on VERO-6 cells and the secondary antibodies used for immunohistochemistry on all studied samples.

| **Primary antibodies** | | | | | |
| --- | --- | --- | --- | --- | --- |
| **Target protein** | **Supplier** | **Reference** | **Source** | **Labelling intensity** | |
|  |  |  |  | **Methanol** | **PFA** |
| ACE-2 | Abcam | ab15348 | Rabbit | +++ | ++ |
| TMPRSS2 | Abcam | ab92323 | Rabbit | ++ | +++ |
| Cathepsin B | SCBT | sc-365558 | Mouse | + | +++ |
| Cathepsin L | SCBT | sc-32320 | Mouse | +++ | +++ |
| SARS-CoV-2 spike | ProSci | 3525 | Rabbit | +++ (cells)  ? (corneas) | +++ (cells)  ? (corneas) |
| SARS-CoV-2 Nucleocapsid | SinoBiological | 40143 | Rabbit | +++ (cells)  ? (corneas) | +++ (cells)  ? (corneas) |
| **Secondary antibodies** | | | | | |
| **Target IgG** | **Fluorochromes** | **Purification method** | | **Supplier** | **Reference** |
| Goat anti-Rabbit IgG | Alexa 555 | Highly Cross-Adsorbed | | Invitrogen | A32732 |
| Goat anti-Rabbit IgG | Alexa 488 | Highly Cross-Adsorbed | | Invitrogen | A11034 |
| Goat anti-Mouse IgG | Alexa 555 | Highly Cross-Adsorbed | | Invitrogen | A32727 |
| Goat anti-Mouse IgG | Alexa 488 | Highly Cross-Adsorbed | | Invitrogen | A32723 |

Legend: + = mild; ++ = moderate; +++ = high; ? = signal specificity unclear/unvalidated; PFA: paraformaldehyde.
