# Supplementary material for: Exploration of the ocular surface infection by SARS-CoV-2 and implications for corneal donation: An ex vivo study
Source: PLoS Med. 2022 Mar 1;19(3):e1003922. doi: 10.1371/journal.pmed.1003922 (PMC8887728; doi:10.1371/journal.pmed.1003922)
Supplement: S2 Table — RT-PCR, reverse transcription PCR; SARS-CoV-2, Severe Acute Respiratory Syndrome Coronavirus 2. (DOCX) [file pmed.1003922.s011.docx]

**S2 Table.** Sequences of primers used for quantitative RT-PCR study of SARS-CoV-2 receptor and activators.

| Protein | Amplicon size (pb) | Forward sequence | Reverse sequence |
| --- | --- | --- | --- |
| Cathepsin L | 184 | TCAGGCAGGTGATGAATGGC | CCTTCAAGAGCACCAGTAGCA |
| Cathepsin B | 135 | GGAGGGAGCTTTCTCTGTGT | CAGTAGGGTGTGCCATTCTC |
| ACE-2 | 197 | TGGTCTTCTGTCACCCGATT | CCCCAACTATCTCTCGCTTCA |
| TMPRSS2 | 111 | GGGGATACAAGCTGGGGTTC | TTAGCCGTCTGCCCTCATTT |
| GAPDH | 172 | GAAGGTGAAGGTCGGAGT | GAAGATGGTGATGGGATTTC |
