# Supplementary material for: Exploration of the ocular surface infection by SARS-CoV-2 and implications for corneal donation: An ex vivo study
Source: PLoS Med. 2022 Mar 1;19(3):e1003922. doi: 10.1371/journal.pmed.1003922 (PMC8887728; doi:10.1371/journal.pmed.1003922)
Supplement: S3 Table — ACE-2, angiotensin-converting enzyme 2. (DOCX) [file pmed.1003922.s012.docx]

**S3 Table.** Raw data of the expression of ACE-2, TMPRSS2, Cathepsins B and L genes in the epithelium of *ex vivo* infected central cornea and corneoscleral rim at H0 (2^-ΔCt^ values) (Fig 3).

| ACE2 | Central cornea | 0,00692479 | 0,00663927 | 0,01014379 | 0,000078006 | 0,01248799 |
| --- | --- | --- | --- | --- | --- | --- |
|  | corneoscleral rim | 0,02428979 | 0,00988349 | 0,03702372 | 0,05264699 | 0,04083434 |
| TMPRSS2 | Central cornea | 0,00033066 | 0,00040573 | 0,00196661 | 0,000062439 | 0,0006259 |
|  | corneoscleral rim | 0,00453555 | 0,0014465 | 0,02782881 | 0,04808668 | 0,00996681 |
| CTSL | Central cornea | 0,00913992 | 0,01448281 | 0,08327332 | 0,00245975 | 0,03659805 |
|  | corneoscleral rim | 0,01868593 | 0,02161608 | 0,07331232 | 0,1129007 | 0,100870 |
| CTSB | Central cornea | 0,02213307 | 0,02015873 | 0,1001144 | 0,00133911 | 0,0388314 |
|  | corneoscleral rim | 0,108010 | 0,06753136 | 0,2552994 | 0,3297895 | 0,09669681 |
