# Supplementary material for: Exploration of the ocular surface infection by SARS-CoV-2 and implications for corneal donation: An ex vivo study
Source: PLoS Med. 2022 Mar 1;19(3):e1003922. doi: 10.1371/journal.pmed.1003922 (PMC8887728; doi:10.1371/journal.pmed.1003922)
Supplement: S4 Table — SARS-CoV-2, Severe Acute Respiratory Syndrome Coronavirus 2. (DOCX) [file pmed.1003922.s013.docx]

**S4 Tables.** Raw data of SARS-CoV-2 RNA expression as mean number of total IP4 copies, of IP4 positive copies (IP4+) and negative copies (IP4-) in the epithelium of central cornea and corneoscleral rim at 30 min (H0) and 24h (H24) after Sars-CoV-2 ex-vivo infection (Fig 4) are provided in the tables below:

| Total IP4 copies | | | |
| --- | --- | --- | --- |
| Central cornea | | Corneoscleral rim | |
| H0 | H24 | H0 | H24 |
| 9,846894e+007 | 9,731404e+008 | 2,854400e+009 | 3,872320e+010 |
| 4,324682e+008 | 7,935985e+008 | 1,563881e+009 | 5,122500e+010 |
| 1,694043e+008 | 7,085946e+009 | 5,769069e+009 | 3,970694e+010 |
| 1,792579e+008 | 6,137281e+009 | 1,182200e+010 | 2,847300e+010 |
| 6,458458e+007 | 5,921721e+009 | 3,849153e+009 | 2,571246e+010 |
| 9,288204e+007 | 1,217893e+009 | 6,650447e+009 | 4,523995e+010 |
| 1,103532e+008 | 8,547324e+008 | 1,510500e+010 | 3,009600e+010 |
| 1,333606e+008 | 3,980516e+009 | 4,535473e+009 | 1,263251e+010 |
| 7,434692e+007 | 2,984852e+009 | 3,424680e+009 | 4,099800e+010 |

| IP4+ strand copies | | | |
| --- | --- | --- | --- |
| Central cornea | | Corneoscleral rim | |
| H0 | H24 | H0 | H24 |
| 2,750035e+007 | 1,583077e+007 | 2,036364e+008 | 1,015946e+009 |
| 7041303,000000 | 8911362,000000 | 1,059977e+008 | 1,210683e+009 |
| 1,094582e+007 | 8,685926e+007 | 3,462643e+008 | 7,829281e+008 |
| 5,609530e+007 | 8,049279e+007 | 2,437979e+008 | 5,247592e+008 |
| 3,238524e+007 | 8,769811e+007 | 3,102160e+008 | 4,603575e+008 |
| 2,159994e+007 | 3,410234e+007 | 4,231862e+008 | 1,066392e+009 |
| 2,985676e+007 | 4,244094e+007 | 9,543428e+008 | 8,172978e+008 |
| 1,947223e+007 | 6,040984e+007 | 3234745,000000 | 1,995613e+008 |
| 1,886991e+007 | 5,135553e+007 | 3,542255e+008 | 9,483542e+007 |

| IP4- strand copies | | | |
| --- | --- | --- | --- |
| Central cornea | | Corneoscleral rim | |
| H0 | H24 | H0 | H24 |
| 6932733,000000 | 2428397,000000 | 4986295,000000 | 2,086108e+008 |
| 3991471,000000 | 2428397,000000 | 4114784,000000 | 1,661562e+008 |
| 2,077524e+007 | 8261672,000000 | 1,089499e+008 | 4,033112e+007 |
| 5904411,000000 | 2472742,000000 | 6,195260e+007 | 1,486130e+007 |
| 2,134883e+007 | 5,182592e+007 | 2,442825e+008 | 1,559335e+007 |
| 1,627026e+007 | 3090338,000000 | 3,725926e+008 | 1,338587e+008 |
| 1,366368e+007 | 1,147158e+007 | 4224000,000000 | 1,801908e+008 |
| 3221929,000000 | 1,451986e+007 | 2,995711e+008 | 7010117,000000 |
| 5155978,000000 | 2428397,000000 | 7,153011e+007 | 3,357326e+007 |
