# Supplementary material for: Exploration of the ocular surface infection by SARS-CoV-2 and implications for corneal donation: An ex vivo study
Source: PLoS Med. 2022 Mar 1;19(3):e1003922. doi: 10.1371/journal.pmed.1003922 (PMC8887728; doi:10.1371/journal.pmed.1003922)
Supplement: S5 Table — Ct, cycle threshold. (DOCX) [file pmed.1003922.s014.docx]

**S5 Table**. Corresponding raw Ct values (S5 Fig):

| PCR Cov-IP4-RdRP | Corneal medium H0 | 32,62 | 29,75 | 32 | 31,27 | 33,51 | 32,61 | 32,19 | 31,87 | 33,22 |
| --- | --- | --- | --- | --- | --- | --- | --- | --- | --- | --- |
|  | Corneal medium H24 | 28,28 | 28,65 | 24,84 | 25,1 | 25,2 | 27,89 | 28,5 | 25,85 | 26,34 |
|  | Corneoscleral medium H0 | 27,39 | 28,45 | 26,16 | 24,9 | 26,88 | 25,9 | 24,49 | 26,62 | 27,09 |
|  | corneoscleral medium H24 | 22,97 | 22,37 | 22,79 | 23,37 | 23,54 | 22,57 | 23,27 | 24,78 | 22,74 |
| PCR Cov-N-2021 | Corneal medium H0 | 29,67 | 32 | 27,82 | 29,29 | 30,48 | 30,85 | 31,25 | 30,61 | 30,64 |
|  | Corneal medium H24 | 30,4 | 31,07 | 27,31 | 26,28 | 27,99 | 31,08 | 30,93 | 28,07 | 29,05 |
|  | Corneoscleral medium H0 | 25,76 | 28,58 | 25,86 | 26,53 | 27,18 | 27,21 | 26,23 | 26,4 | 26,98 |
|  | corneoscleral medium H24 | 23,95 | 24,35 | 24,35 | 25,34 | 26 | 25,37 | 26,4 | 26,87 | 25,41 |
| PCR Cov-RdRP-2021 | Corneal medium H0 | 28,11 | 30,05 | 26,95 | 27,93 | 28,98 | 28,96 | 29,43 | 28,79 | 28,97 |
|  | Corneal medium H24 | 28,85 | 29,04 | 26 | 25,46 | 26,74 | 29,43 | 28,9 | 26,89 | 27,7 |
|  | Corneoscleral medium H0 | 25,04 | 27 | 24,94 | 25,35 | 25,96 | 25,7 | 24,96 | 25,33 | 25,77 |
|  | corneoscleral medium H24 | 23,17 | 23,31 | 23,32 | 24,28 | 25,11 | 24,17 | 25,16 | 25,86 | 24,26 |
