# Supplementary material for: Exploration of the ocular surface infection by SARS-CoV-2 and implications for corneal donation: An ex vivo study
Source: PLoS Med. 2022 Mar 1;19(3):e1003922. doi: 10.1371/journal.pmed.1003922 (PMC8887728; doi:10.1371/journal.pmed.1003922)
Supplement: S6 Table — Ct, cycle threshold; RT-qPCR, reverse transcription quantitative PCR. (DOCX) [file pmed.1003922.s015.docx]

**Table S6**: Ct values obtained by RT-qPCR for the panel of samples extracted with the two extraction kits.

| Sample | RNeasy mini-Kit (Qiagen) | Quick-RNA kit (Zymo Research) |
| --- | --- | --- |
| Viral RNA 10^4^ | 32.1776941 | 31.32989758 |
| Viral RNA 10^5^ | 30.30301716 | 28.92803739 |
| Viral RNA 10^6^ | 25.74398481 | 24.20742088 |
| Viral RNA 10^7^ | 22.02098001 | 20.50049656 |
| Viral RNA 10^8^ | 18.12406037 | 16.99410453 |
| Viral RNA 10^9^ | 15.51126642 | 13.82675612 |
| Viral RNA 10^10^ | 10.77337875 | 9.266310925 |
| Positive control 1 | 27.76203747 | 27.06755742 |
| Positive control 2 | 28.22990972 | 28.28131659 |
| Negative control 1 | Undetermined | Undetermined |
| Negative control 2 | Undetermined | Undetermined |
| Negative control (no sample) | Undetermined | Undetermined |
| Negative control (water without reaction mix) | Undetermined | Undetermined |
