# Supplementary material for: Exploration of the ocular surface infection by SARS-CoV-2 and implications for corneal donation: An ex vivo study
Source: PLoS Med. 2022 Mar 1;19(3):e1003922. doi: 10.1371/journal.pmed.1003922 (PMC8887728; doi:10.1371/journal.pmed.1003922)
Supplement: Attachment — Submitted filename: Response_to_reviewers_R1.docx [file pmed.1003922.s016.docx]

**COVER LETTER**

Saint-Etienne, December 8^th^, 2021

Dear Editor,

Please find attached a revised version of our article titled “**Exploration of the ocular surface infection by SARS-CoV-2 and implications for corneal donation.** **An *ex vivo* study**” (PMEDICINE-D-21-02421R1)

We have carefully considered and responded to all the points addressed by the reviewers.

As per your instructions, all amendments in the revised version are stated in our point-by-point response and are marked in red in the article.

We greatly hope that this new version will meet the reviewers' expectations and comply with your editorial policy.

Yours sincerely,

Professor Gilles THURET, MD, PhD

Saint-Etienne University Hospital

"Corneal Graft Biology, Engineering, and Imaging" Laboratory EA 2521, SFR143

Faculty of Medicine

Saint-Etienne

France

Institut Universitaire de France, Paris

Guidelines for revised paper: <http://journals.plos.org/plosmedicine/s/revising-your-manuscript>

**Requests from the editors**:

1. Requests of Reviewers: Please completely address all points raised by reviewers. In particular, please address Reviewer 2’s request to clarify/share the SARS-CoV-2 isolation/culture methods, the verification of ex vivo infection using an additional virus-specific sequence, the question of potential for false positive results and whether tissue contains replicable virus, and please address Reviewer 3’s point that the data do not support the conclusion that replication of SARS-CoV-2 in tissue was demonstrated.

2. Title: Please revise your title according to PLOS Medicine's style. Your title must be nondeclarative and not a question. It should begin with main concept if possible. "Effect of" should be used only if causality can be inferred, i.e., for an RCT. Please place the study design ("A randomized controlled trial," "A retrospective study," "A modelling study," etc.) in the subtitle (ie, after a colon).

We have modified the title accordingly:

**Exploration of the ocular surface infection by SARS-CoV-2 and implications for corneal donation: an *ex vivo* study.**

3. Data Availability Statement: Please revise the data statement, which now reads: “All relevant data are within the manuscript and its Supporting Information files.”

PLOS Medicine requires that the de-identified data underlying the specific results in a published article be made available, without restrictions on access, in a public repository or as Supporting Information at the time of article publication, provided it is legal and ethical to do so. Please see the policy at

<http://journals.plos.org/plosmedicine/s/data-availability>

and FAQs at

<http://journals.plos.org/plosmedicine/s/data-availability#loc-faqs-for-data-policy>
PLOS defines the “minimal data set” to consist of the data set used to reach the conclusions drawn in the manuscript with related metadata and methods, and any additional data required to replicate the reported study findings in their entirety. Authors do not need to submit their entire data set, or the raw data collected during an investigation. However, we request that you please make available the values behind the means, standard deviations and other measures reported, any values derived from images, and the values used to build graphs.
For each data source used in your study:

a) If the data are freely or publicly available, note this and state the location of the data: within the paper, in Supporting Information files, or in a public repository (include the DOI or accession number).

b) If the data are owned by a third party but freely available upon request, please note this and state the owner of the data set and contact information for data requests (web or email address). Note that a study author cannot be the contact person for the data.

c) If the data are not freely available, please describe briefly the ethical, legal, or contractual restriction that prevents you from sharing it. Please also include an appropriate contact (web or email address) for inquiries (again, this cannot be a study author).

Our data are freely available and the raw data files relating to the various graphical representations have been added in the Supporting Information file and are reproduced thereafter:

1/ SARS-CoV-2 RNA expression as mean number of total IP4 copies, of IP4 positive copies (IP4+) and negative copies (IP4-) in the epithelium of central cornea and corneoscleral rim at 30 min (H0) and 24h (H24) after Sars-CoV-2 *ex vivo* infection (data of Figure 4).

| Total IP4 copies | | | |
| --- | --- | --- | --- |
| Central cornea | | corneoscleral rim | |
| H0 | H24 | H0 | H24 |
| 9,846894e+007 | 9,731404e+008 | 2,854400e+009 | 3,872320e+010 |
| 4,324682e+008 | 7,935985e+008 | 1,563881e+009 | 5,122500e+010 |
| 1,694043e+008 | 7,085946e+009 | 5,769069e+009 | 3,970694e+010 |
| 1,792579e+008 | 6,137281e+009 | 1,182200e+010 | 2,847300e+010 |
| 6,458458e+007 | 5,921721e+009 | 3,849153e+009 | 2,571246e+010 |
| 9,288204e+007 | 1,217893e+009 | 6,650447e+009 | 4,523995e+010 |
| 1,103532e+008 | 8,547324e+008 | 1,510500e+010 | 3,009600e+010 |
| 1,333606e+008 | 3,980516e+009 | 4,535473e+009 | 1,263251e+010 |
| 7,434692e+007 | 2,984852e+009 | 3,424680e+009 | 4,099800e+010 |

| IP4+ strains copies | | | |
| --- | --- | --- | --- |
| Central cornea | | corneoscleral rim | |
| H0 | H24 | H0 | H24 |
| 2,750035e+007 | 1,583077e+007 | 2,036364e+008 | 1,015946e+009 |
| 7041303,000000 | 8911362,000000 | 1,059977e+008 | 1,210683e+009 |
| 1,094582e+007 | 8,685926e+007 | 3,462643e+008 | 7,829281e+008 |
| 5,609530e+007 | 8,049279e+007 | 2,437979e+008 | 5,247592e+008 |
| 3,238524e+007 | 8,769811e+007 | 3,102160e+008 | 4,603575e+008 |
| 2,159994e+007 | 3,410234e+007 | 4,231862e+008 | 1,066392e+009 |
| 2,985676e+007 | 4,244094e+007 | 9,543428e+008 | 8,172978e+008 |
| 1,947223e+007 | 6,040984e+007 | 3234745,000000 | 1,995613e+008 |
| 1,886991e+007 | 5,135553e+007 | 3,542255e+008 | 9,483542e+007 |

| IP4- strains copies | | | |
| --- | --- | --- | --- |
| Central cornea | | corneoscleral rim | |
| H0 | H24 | H0 | H24 |
| 6932733,000000 | 2428397,000000 | 4986295,000000 | 2,086108e+008 |
| 3991471,000000 | 2428397,000000 | 4114784,000000 | 1,661562e+008 |
| 2,077524e+007 | 8261672,000000 | 1,089499e+008 | 4,033112e+007 |
| 5904411,000000 | 2472742,000000 | 6,195260e+007 | 1,486130e+007 |
| 2,134883e+007 | 5,182592e+007 | 2,442825e+008 | 1,559335e+007 |
| 1,627026e+007 | 3090338,000000 | 3,725926e+008 | 1,338587e+008 |
| 1,366368e+007 | 1,147158e+007 | 4224000,000000 | 1,801908e+008 |
| 3221929,000000 | 1,451986e+007 | 2,995711e+008 | 7010117,000000 |
| 5155978,000000 | 2428397,000000 | 7,153011e+007 | 3,357326e+007 |

2/ Expression of ACE-2, TMPRSS2, Cathepsins B and L genes in the epithelium of *ex vivo* infected central cornea and corneoscleral rim at H0 (2^-ΔCt^) (data of figure 3):

| ACE2 | Central cornea | 0,00692479 | 0,00663927 | 0,01014379 | 0,000078006 | 0,01248799 |
| --- | --- | --- | --- | --- | --- | --- |
|  | corneoscleral rim | 0,02428979 | 0,00988349 | 0,03702372 | 0,05264699 | 0,04083434 |
| TMPRSS2 | Central cornea | 0,00033066 | 0,00040573 | 0,00196661 | 0,000062439 | 0,0006259 |
|  | corneoscleral rim | 0,00453555 | 0,0014465 | 0,02782881 | 0,04808668 | 0,00996681 |
| CTSL | Central cornea | 0,00913992 | 0,01448281 | 0,08327332 | 0,00245975 | 0,03659805 |
|  | corneoscleral rim | 0,01868593 | 0,02161608 | 0,07331232 | 0,1129007 | 0,100870 |
| CTSB | Central cornea | 0,02213307 | 0,02015873 | 0,1001144 | 0,00133911 | 0,0388314 |
|  | corneoscleral rim | 0,108010 | 0,06753136 | 0,2552994 | 0,3297895 | 0,09669681 |

4. Throughout: Please include line numbers with the revised version.

The final version of the revised article now includes line numbers.

5. Throughout: We suggest referring to “donors diagnosed with COVID-19” or similar rather than “COVID19 donors” or “COVID-19 deceased donors” throughout the text.

“COVID-19 donors” and “non-COVID-19 donors” were abbreviations selected to facilitate writing, reading and understanding of the experimental part (methods and results). However, these terms were not correctly defined in the beginning of the material section. We updated the text accordingly (line 200-204). Furthermore, we have ensured that these abbreviations are not used in the remaining text (abstract, introduction and discussion) and replaced them by “donors diagnosed with COVID-19”.

6. Abstract: Methods and Findings: Please indicate the setting of the study and describe the cornea donor population/source of donor tissue and clearly note outcome measures for each part of the study.

The final version was updated accordingly. The new text is as below:

**“Methods and Findings**

1/ The expression pattern of SARS-CoV-2 receptor ACE-2 and activators TMPRSS2 and Cathepsins B and L in ocular surface tissues from non-affected donors was explored by immunohistochemistry (n = 10 corneas, 78 ± 11 years, 40% female) and qPCR (n = 5 corneas, 80 ± 12 years, 40% female). 2/ Five freshly excised corneas (80 ± 12 years, 40% female) were infected ex vivo with highly concentrated SARS-CoV-2 solution (10^7^ TCID_50_/mL). Viral RNA was extracted from tissues and culture media and quantified by RT-qPCR (viral RNA copies) 30 min (H0) and 24h (H24) after infection. 3/ SARS-CoV-2 RNA was tested by RT-qPCR (Ct value) in both corneas and organ culture media from 14 donors diagnosed with COVID-19 (74 ± 10 years, 29% female) and 26 healthy donors (79 ± 13 years, 57% female), and in organ culture media only from 133 consecutive non-affected donors from 2 eye banks (73 ± 13 years, 29% female).

The expression of receptor and activators was variable among samples at both protein and mRNA level. ACE-2 was localized mainly in the most superficial epithelial cells of peripheral cornea, limbus and conjunctiva, whereas TMPRSS2 was mostly expressed in all layers of bulbar conjunctiva.

A significant increase in total and positive IP4 strands was observed 24h after experimental infection in central cornea (1.1x10^8^ [95% CI: 6.4x10^7^ – 2.4x10^8^] to 3.0x10^9^ [1.4x10^9^ – 5.3x10^9^], p = 0.0039 and 2.2x10^7^ [1.4x10^7^ – 3.6x10^7^] to 5.1x10^7^ [2.9x10^7^ – 7.5x10^7^], p = 0.0117, respectively) and in corneoscleral rim (4.5x10^9^ [2.7x10^9^ – 9.6x10^9^] to 3.9x10^10^ [2.6x10^10^ – 4.4x10^10^], p = 0.0039 and 3.1x10^8^ [1.2x10^8^ – 5.3x10^8^] to 7.8x10^8^ [3.9x10^8^ – 9.9x10^8^], p = 0.0391, respectively). Viral RNA copies in *ex vivo* corneas were highly variable from one donor to another. Finally, viral RNA was detected in 3 out of 28 corneas (11%) from donors diagnosed with COVID-19. All samples from the 159 non-affected donors were negative for SARS-CoV-2 RNA. The main limitation of this study relates to the limited sample size, due to limited access to donors diagnosed with COVID-19 and concomitant decrease in the procurement corneas from non-affected donors.”

7. Abstract: Methods and Findings: Please quantify the main results (with 95% CIs and p values).
We added a sentence with the quantification of the main results in this section of the abstract (L74-77, see above) and in the results section (L469-476).

8. Abstract: Conclusions: Please address the study implications without overreaching what can be concluded from the data; beginning with the phrase "In this study, we observed ..." may be useful.

We have updated the abstract conclusion accordingly (L84-88). The abstract conclusion is now:

“In this study we observed a significant expression of SARS-CoV-2 receptors and activators at the human ocular surface and a variable increase in viral RNA copies 24h after experimental infection of freshly excised human corneas. We also found viral RNA only in a very limited percentage of donors with positive nasopharyngeal PCR. The low rate of positivity in donors diagnosed with COVID-19 calls into question the utility of donor selection algorithms.”

9. Author summary: We ask that you include a short, non-technical Author Summary of your research to make findings accessible to a wide audience that includes both scientists and non-scientists. The Author Summary should immediately follow the Abstract in your revised manuscript. This text is subject to editorial change and should be distinct from the scientific abstract. Please see our author guidelines for more information: <https://journals.plos.org/plosmedicine/s/revising-your-manuscript#loc-author-summary>

We added the following Author Summary after the abstract:

**-**Why Was This Study Done?

Corneal transplantation is by far the most common transplantation procedure in the world and there is a severe lack of corneal donation to respond to the increasing patient need. The persistent COVID-19 pandemic has dramatically worsened this situation by limiting the access to corneas during a long period of the pandemic and finally increasing the long list of contraindications to corneal donation based solely on an exacerbated precautionary principle.

Exploration of the SARS-CoV-2 risk of transmission through corneal transplantation is mandatory to adapt the donor selection strategy and improve corneal tissue procurement in the persisting COVID-19 crisis.

-What Did the Researchers Do and Find?

We explored and observed the expression of 4 proteins related to molecular pathways allowing SARS-CoV-2 cellular entry at the ocular surface (cornea, bulbar conjunctiva, 10 samples). These proteins were differentially located in the studied tissues and their expression was variable among donors.

The experimental infection of five freshly excised corneas with highly concentrated SARS-CoV-2 viral solution promoted only moderate and variable viral RNA multiplication in corneal samples.

Twenty-four hours after inoculation, a significant increase in total and positive strand viral RNA was observed in the epithelium of central cornea (p-value: 0.0039 for and 0.0117 respectively) and in corneoscleral rim (p-value: 0.0039 and 0.0391 respectively).

We explored the presence of SARS-CoV-2 viral genetic material in corneas and respective storage media from donors tested positive or negative to COVID-19 PCR on nasopharyngeal swab. All samples from the 159 donors not contaminated by COVID-19 were negative for SARS-CoV-2 RNA. SARS-CoV-2 RNA was detected in only 3 corneas out of 28 corneas (11%) from donors diagnosed with COVID-19.

-What Do These Findings Mean?

Our results support that the ocular surface expresses in a variable way the proteins involved in SARS-CoV-2 cellular entry and infection. We observed a moderate and variable increase in total and positive viral RNA after experimental infection of human freshly excised corneas. A very low rate of positivity in donors diagnosed with COVID-19 was observed, supporting a low risk of SARS-CoV-2 presence in donor corneas. This work suggests that it would be beneficial to procure corneas from donors tested positive to COVID-19 (nasopharyngeal swabs), test their corneal storage media and transplant the negative corneas.

10. Methods: Study 1: Please describe the relevant demographics of the 10 cornea donors who did not have COVID-19, and please describe how it was established that they did not have COVID-19. Please describe how these were sampled, and why numbers of IHC samples differed for each marker.

The demographics of the 10 donors were provided in the results but not the method section. This has been updated (L210). The sampling procedure is described L215: “Corneas were procured at a mean of 11 ± 6 hours after death following standard procedure without disinfection to allow preservation of epithelial layers”.

Among the 10 samples examined, 3 corneas from 3 donors were analyzed for another project shortly prior to the start of this study for the expression of ACE-2 (n = 3) and TMPRSS2 (n = 2) and other markers. For these samples, CTSL/B exploration was not possible.

After the study began, 7 other donors were procured to complete the preliminary IHC results and to also explore cathepsin B and L expression. These donors presented a negative PCR on post-mortem nasopharyngeal swab and no clinical signs related to COVID-19 before death. We updated the text accordingly to clarify this aspect (L213-215). Due to a technical issue, CTSB labelling from one sample was not included in the final results.

For the conjunctival epithelial samples, please describe how the 11 healthy donors were selected, and how it was established that they did not have COVID-19.

The donors for conjunctival epithelial samples were healthy patients with normal cornea and conjunctiva, simply undergoing cataract surgery. The donors presented a negative PCR 48 to 72h prior to sampling procedure and no clinical signs related to COVID-19 prior to sampling procedure. This information has been added in line 220-222:

“A total of 11 conjunctival epithelial samples were collected in healthy donors (Mean age 53 ± 24 years, 55% female, patients undergoing cataract surgery and presenting healthy cornea and conjunctiva) by impression cytology using a dedicated device (Eyeprim OS1, OPIA Technologies, Paris, France) and immediately fixed at room temperature during 15 min in 0.5% PFA. Each of the patient had a negative PCR 24 to 48h prior to surgery and were asymptomatic.”

11. Methods: Study 2: Please describe how it was established the 5 donors did not have COVID-19.
The 5 donors presented a negative PCR on post-mortem nasopharyngeal swabs and no clinical signs related to COVID-19 before death, as added in L272-273.

12. Discussion: Please present and organize the Discussion as follows: a short, clear summary of the article's findings; what the study adds to existing research and where and why the results may differ from previous research; strengths and limitations of the study; implications and next steps for research, clinical practice, and/or public policy; one-paragraph conclusion.

We re-organized the discussion following these recommendations and hope it will ease the reading and understanding of this section.

13. Discussion: “We describe for the first time the expression pattern of cathepsins B and L proteins and mRNA at the healthy human ocular surface…” Please temper all claims of primacy with “To the best of our knowledge” or similar.

The text has been updated accordingly (L533).

14. S1 Table: Where label intensity is described, please clarify “?” for SARS-CoV-2 labelling in cornea.

The legend of S1 Table has been completed with a clear legend of intensity grading scheme. (L34 in S1_File)

15. Reporting Guidelines: Please ensure that the study is reported according to the STROBE guideline, or the most relevant guideline for your study, and include the completed STROBE checklist as Supporting Information. When completing the checklist, please use section and paragraph numbers, rather than page numbers. Please add the following statement, or similar, to the Methods: "This study is reported as per the Strengthening the Reporting of Observational Studies in Epidemiology (STROBE) guideline (S1 Checklist)."
Please report your study according to the relevant guideline, which can be found here: <http://www.equator-network.org/>
We ensured the report of the results follows the STROBE guidelines. We have filled and added the STROBE checklist as supplemental information (L2 in S1_File).

Comments from the reviewers:

**Reviewer #1**: The aim of this study is to assess the capacity of human cornea to be infected by SARSCoV-2 and promote its replication ex vivo, and to evaluate the real-life risk of corneal contamination by detecting SARS-CoV-2 RNA in corneas and associated tissues retrieved in COVID-19 and non-COVID-19 donors.

**Comments**:

1- Did the authors undertake an a priori sample size calculation?

As well described (**Beck et al., 2013**), sample size calculation based on software programs is one of the factors affecting the sensitivity of an experiment. In the context of our study, a sample size calculation for the *ex vivo* experiment (study 2) was performed using G Power software v3.1.

1/ *Ex vivo* experimental infection of fresh corneas with SARS-CoV-2 (study 2)

We originally based our study on the results obtained from a published article that performed *in vitro* infection with SARS-CoV-2 virus (**Chu et al., 2020**). Twenty-four hours after inoculation, an increase in total copy number from approximately 5.10^7 copies/ml to 5.10^8 copies/ml was reported. We approximated the standard deviation at half the mean (2.5.10^7 and 2.5x10^8 copies/mL respectively). With an α= 0.05 and β=0.95, we obtained then an effect size of 1.8869 and thereafter a total sample size of 5.

Besides, due to the strict restrictions in corneal retrieval for both research and clinical purposes, no more than five donors fitting inclusion criteria could be included for the *ex vivo* experimentation during the whole study period. Inclusion criteria were strictly followed to maximize homogeneity of the samples (post-mortem delay below 12 h, corneal epithelium integrity, only pairs of corneas from a single donor).

2/ SARS-CoV-2 RNA detection in the ocular surface tissues from COVID-19 and non-COVID-19 donors (Study 3)

In this context (almost no access to patients deceased with COVID-19 clinical signs and very low number of corneas procured in patients tested positive to COVID-19) and due to the descriptive nature of the study 3 (presence/absence of viral RNA in the tested samples), no sample size calculation was performed for this part of the experiments. We included all the available samples during the study period.

**References:**

Beck TW. The importance of a priori sample size estimation in strength and conditioning research. J Strength Cond Res. 2013 Aug;27(8):2323-37. doi: 10.1519/JSC.0b013e318278eea0.

Chu H, Chan JF, Yuen TT, Shuai H, Yuan S, Wang Y, Hu B, Yip CC, Tsang JO, Huang X, Chai Y, Yang D, Hou Y, Chik KK, Zhang X, Fung AY, Tsoi HW, Cai JP, Chan WM, Ip JD, Chu AW, Zhou J, Lung DC, Kok KH, To KK, Tsang OT, Chan KH, Yuen KY. Comparative tropism, replication kinetics, and cell damage profiling of SARS-CoV-2 and SARS-CoV with implications for clinical manifestations, transmissibility, and laboratory studies of COVID-19: an observational study. Lancet Microbe. 2020 May;1(1):e14-e23. doi: 10.1016/S2666-5247(20)30004-5. Epub 2020 Apr 21. PMID: 32835326; PMCID: PMC7173822.

"Data are expressed as Mean ± Standard Deviation (SD). Analysis was performed using GraphPad Prism (GraphPad Software, La Jolla, CA, USA). IP4 total number of copies were compared among groups using paired-T-test. Correlation between gene expression of the various receptor/activators and total IP4 level at H0 was studied using Spearman correlation coefficient."
Can the authors please confirm if the assumption of normality has been verified?

We indeed tested the assumption of normality using the Shapiro-Wilk and Kolmogorov-Smirnov normality tests before performing the statistical analysis. All the data are normally distributed except values of IP4 total in central corneas at H0 and IP4 negative strands in central corneas at H24.

Taking into account these results and the small sample size of the study, we also performed comparisons using non-parametric tests (Wilcoxon Signed Ranks Test for paired samples). To note, the results of the Wilcoxon test are concordant with the results of the parametric T-test and are as follow:

| Wilcoxon | | | Paired t-test | | |
| --- | --- | --- | --- | --- | --- |
|  | p-value | Significativity |  | p-value | Significativity |
| Total Cornea  H0 vs H24 | 0.0039 | ** | Total Cornea  H0 vs H24 | 0.0059 | ** |
| Total Sclera  H0 vs H24 | 0.0039 | ** | Total Sclera  H0 vs H24 | 0.0002 | *** |
| Strand + Cornea H0 vs H24 | 0.0117 | * | Strand + Cornea H0 vs H24 | 0.0176 | * |
| Strand + Sclera  H0 vs H24 | 0.0391 | * | Strand + Sclera  H0 vs H24 | 0.0404 | * |

Data have been updated accordingly in the different concerned sections (results, abstract) and the statistical analysis section has been updated as follows (L407):

“Analysis was performed using GraphPad Prism (GraphPad Software, La Jolla, CA, USA). The normality of the distribution of continuous variables was tested by the Shapiro-Wilk normality test. Continuous variables with normal distribution were reported as Mean ± Standard Deviation (SD); non-normal variables were reported as Median (IQR: InterQuartile Range). Experimental groups were either compared by paired t-test (normal distribution) or Wilcoxon signed-rank test (non-normal distribution).”

Overall, this manuscript presents an important and timely hypothesis generation study (due to the limited sample size, which the authors appropriately acknowledge in the discussion).

**Reviewer #2**: The authors present a study addressing the question of the possibility of a transmission of SARS-CoV-2 via donor corneas. The study is methodologically extensive and deals first with the presence of specific SARS-CoV-2 receptors in corneal and conjunctival tissue, second with the question of whether corneal and conjunctival tissue can be infected with SARS-CoV-2 ex vivo, and third with the occurrence of SARS -CoV-2 positive donor corneas and medium in clinical routine.

The question, if SARS-CoV-2 transmission can take place via corneal transplantation, is of very high importance. Especially regarding the worldwide shortage of transplants and the continuation of the activities of the corneal banks in times of the pandemic. The present study adds valuable information to the current debate. However, the study in its present form falls short of clearly supporting the conclusions drawn. To enhance the present paper to a publishable level, additional experimental data should be included, as well as a thorough revision of the manuscript. Major points:

- **Why didn't the authors employ a commercially available kit of SARS-CoV-2?**

When the study started, no RT-PCR kits were commercially available and only one validated PCR method was available and used by our university hospital virology platform. This PCR, developed and validated by Institut Pasteur, was at this time considered as a reference by the scientific community in France (**Haddar et al., 2020**), and further validated and extensively used by our collaborators at the University hospital of St Etienne. Indeed, a study rapidly showed the high sensitivity and specificity of this technique at this time (**Etievant et al., 2020**).

**References:**

Haddar C, Verhoeven PO, Bourlet T, Pozzetto B, Pillet S. Brief comparative evaluation of six open one-step RT-qPCR mastermixes for the detection of SARS-CoV-2 RNA using a Taqman probe. J Clin Virol. 2020 Nov;132:104636. doi: 10.1016/j.jcv.2020.104636. Epub 2020 Sep 8. PMID: 33099260; PMCID: PMC7476897.

Etievant S, Bal A, Escuret V, Brengel-Pesce K, Bouscambert M, Cheynet V, Generenaz L, Oriol G, Destras G, Billaud G, Josset L, Frobert E, Morfin F, Gaymard A. Performance Assessment of SARS-CoV-2 PCR Assays Developed by WHO Referral Laboratories. J Clin Med. 2020 Jun 16;9(6):1871. doi: 10.3390/jcm9061871. PMID: 32560044; PMCID: PMC7355678.

-The present approach makes this study essentially irreproducible for other groups and thus possibly does not meet PLOS ONE's publication criteria as outlined on the journal's website.

**Please make this isolation & culture method public using the supporting information section**. Which variant of SARS-CoV-2 was isolated and successfully cultured?

We thank the reviewer for pointing out this important lack in the methodology description that indeed would block the reproduction of our results by other teams.

The SARS-CoV-2 strain used in this study is the RoBO strain, a clinical isolate obtained from a nasopharyngeal aspirate of a patient hospitalized at the university hospital of Saint-Etienne for a severe COVID-19. The strain was isolated and cultured on Vero-E6 cells. Robo is a “Wuhan” 2019 type strain which belong to the 19A lineage and was used in other published studies from Saint-Etienne University (**Legros et al., 2021**)

We have thus updated the method section of study 2 with the relevant information and the reference of the complete protocol (L280):

“The viral strain (RoBo strain) used in this study was a clinical isolate obtained from a nasopharyngeal aspirate of a patient hospitalized at the University Hospital of Saint-Etienne for severe COVID-19 and cultured on Vero-E6 cells (CRL-1586, ATCC) (**Legros et al., 2021**).”

**Reference:**

Legros V, Denolly S, Vogrig M, Boson B, Siret E, Rigaill J, Pillet S, Grattard F, Gonzalo S, Verhoeven P, Allatif O, Berthelot P, Pélissier C, Thiery G, Botelho-Nevers E, Millet G, Morel J, Paul S, Walzer T, Cosset FL, Bourlet T, Pozzetto B. A longitudinal study of SARS-CoV-2-infected patients reveals a high correlation between neutralizing antibodies and COVID-19 severity. Cell Mol Immunol. 2021 Feb;18(2):318-327. doi: 10.1038/s41423-020-00588-2. Epub 2021 Jan 6. PMID: 33408342; PMCID: PMC7786875.

- **The proof of a successful infection ex vivo was only provided via the RdRp gene. The authors should use at least one additional virus-specific sequence (e.g. nucleocapsid protein gene or S-gene) for PCR detection to support the result**. SEM and TEM images as well as immunostaining did not.

The reviewer highlighted a crucial point concerning the complexity of studying viral RNA multiplication during the *ex vivo* infection. According to your suggestion, we have performed a new qPCR targeting both the N gene and RdRp gene of SARS-CoV-2 (PCR called Cov-N-2021 and Cov-RdRp-2021 respectively) on all the remaining samples (*ex vivo* infection media, n = 9 samples for corneas and scleral rims at both H0 and H24) and, this time, using a commercial kit (ARGENE SARS-CoV-2 R-GENE kit, #423735, Biomérieux) as described by **Ferté et al.**, **2021**. As the RNA extracts of the epithelia of *ex vivo* infected corneas were used to carry out the exploration of the SARS-CoV-2 receptors genes by qPCR, no remaining samples were available to perform these new PCRs.

The results of the new PCRs (Fig SX B and C, Cov-N-2021 PCR and Cov-RdRp-2021 respectively), confirmed those obtained with the IP4 PCR (Fig SX A, Cov-IP4-RdRp-2020) with a significant increase in total RNA in the corneal and in the corneoscleral rim media 24 h after SARS-CoV-2 infection.

As Ct values from each data series were normally distributed (Shapiro-Wilk), a T-test was performed to compare the mean Ct of corneal medium and corneoscleral medium at H0 and H24. Graphical results with p-values have been added as supplemental figure (S5), raw data added to supporting information, and the main text of result section was properly modified (L480):

“Comparable results were obtained with the amplification of both RdRp and N genes in culture media using a commercially available kit (S5 Fig).”

**S5 Figure**: SARS-CoV-2 RNA expression as mean Ct ± SD of Cov-IP4-RdRp-2020 PCR (A), of Cov-N-2021 PCR (B) and of Cov-RdRp-2021 PCR (C) in the incubation media of central cornea and corneoscleral rim at 30 min (H0) and 24h (H24) after SARS-CoV-2 *ex vivo* infection. Results are provided for the respective culture medium of central cornea and corneoscleral rim.

Corresponding Ct values (S5 Figure):

| PCR Cov-IP4-RdRP | Corneal medium H0 | 32,62 | 29,75 | 32 | 31,27 | 33,51 | 32,61 | 32,19 | 31,87 | 33,22 |
| --- | --- | --- | --- | --- | --- | --- | --- | --- | --- | --- |
|  | Corneal medium H24 | 28,28 | 28,65 | 24,84 | 25,1 | 25,2 | 27,89 | 28,5 | 25,85 | 26,34 |
|  | Corneoscleral medium H0 | 27,39 | 28,45 | 26,16 | 24,9 | 26,88 | 25,9 | 24,49 | 26,62 | 27,09 |
|  | corneoscleral medium H24 | 22,97 | 22,37 | 22,79 | 23,37 | 23,54 | 22,57 | 23,27 | 24,78 | 22,74 |
| PCR Cov-N-2021 | Corneal medium H0 | 29,67 | 32 | 27,82 | 29,29 | 30,48 | 30,85 | 31,25 | 30,61 | 30,64 |
|  | Corneal medium H24 | 30,4 | 31,07 | 27,31 | 26,28 | 27,99 | 31,08 | 30,93 | 28,07 | 29,05 |
|  | Corneoscleral medium H0 | 25,76 | 28,58 | 25,86 | 26,53 | 27,18 | 27,21 | 26,23 | 26,4 | 26,98 |
|  | corneoscleral medium H24 | 23,95 | 24,35 | 24,35 | 25,34 | 26 | 25,37 | 26,4 | 26,87 | 25,41 |
| PCR Cov-RdRP-2021 | Corneal medium H0 | 28,11 | 30,05 | 26,95 | 27,93 | 28,98 | 28,96 | 29,43 | 28,79 | 28,97 |
|  | Corneal medium H24 | 28,85 | 29,04 | 26 | 25,46 | 26,74 | 29,43 | 28,9 | 26,89 | 27,7 |
|  | Corneoscleral medium H0 | 25,04 | 27 | 24,94 | 25,35 | 25,96 | 25,7 | 24,96 | 25,33 | 25,77 |
|  | corneoscleral medium H24 | 23,17 | 23,31 | 23,32 | 24,28 | 25,11 | 24,17 | 25,16 | 25,86 | 24,26 |

References :

Ferté T, Ramel V, Cazanave C, Lafon ME, Bébéar C, Malvy D, Georges-Walryck A, Dehail P. Accuracy of COVID-19 rapid antigenic tests compared to RT-PCR in a student population: The StudyCov study. J Clin Virol. 2021 Aug;141:104878. doi: 10.1016/j.jcv.2021.104878. Epub 2021 Jun 5. PMID: 34134035; PMCID: PMC8178956.

- The nomenclature of contamination and infection in this study is confusing (e.g. page 5, para 3: "Study 2"). In the abstract it is stated, that "the aim of the study is … capacity of human cornea to be infected by SARS-CoV-2 …". As I understand it, it is infection of corneal tissue and not contamination in Study 2. Please specify and change the manuscript accordingly.

As rightly pointed out, the use of the two terms “infection” and “contamination” is not clear. In study 2, it is indeed an exploration of the possible infection of cornea. We thus updated the use of this term for this purpose and kept the term “contamination” to describe the presence of viral RNA in the human samples tested in study 3 (presence of a viral material in a human tissue).

- P 7, para 2: A Ct value of 40 as threshold value is unusually high leading to a potential high false positive rate. As the authors rightly point out in the discussion (page 16, para 1), the question of replicable viruses in corneal tissue is crucial for a potential risk to corneal recipients.

This is indeed a very pertinent question raised by the reviewer and probably motivated by the unclear description of this aspect of PCR settings. The maximal number of PCR cycles obtained for the viral RNA amplification was 40, for both H0 and H24 conditions. Ct values ranged from 20 to 30 for total IP4, from 20 to 38 for IP4+ strand and from 25 to 40 for IP4- strand. This information has been added L346 in the method section. The highest Ct values relate to the low viral load observed at H0.

- The authors did not test, if the infected tissue (Study 2) contained replicable virus, this should be additionally performed.

We thank the reviewer for this important remark concerning one limitation of the study. To further explore the production of infectious viral particles, we cultivated Vero-E6 cells in the presence of organ culture media (CorneaMax) sampled from the *ex vivo* infections at H0 and H24 and kept frozen for 16 months at -80°C, as it was the only remaining experimental material available to perform this experiment.

**Methods**:

To evaluate the production of SARS-CoV-2 infectious particles during the *ex vivo* experiment (study2), we incubated 200 µl of organ culture media (retrieved after the 24 h-incubation period and stored during 16 months at -80°C) with 80% confluent Vero-E6 cells in our BSL3 platform (n=9 H24 central cornea media and n=9 H24 corneoscleral rim media). Additionally, H0 central cornea (n=9) and H0 corneoscleral rim media (n = 9) sampled at (retrieved before the 24 h-incubation period)

Negative control cells were incubated with sterile culture medium whereas positive control cells were incubated with the viral solution used for the infection procedure (undiluted : 10^7^ TCID50/mL and diluted 1/100e : 10^5^ TCID50/mL). Cells were incubated 7 days at 37°C and 5% CO2 and daily visually checked to observe the presence of cytopathogenic effect caused by SARS-CoV-2 multiplication. At the end of incubation period, cells were then fixated in 0.5% PFA and immunolabeled against SARS-CoV-2 Nucleocapsid protein (40150 R007, SinoBiological), nuclei were stained with TO-PRO™-3 Iodide (T3605, Invitrogen). Cells were observed by confocal laser scanning microscopy (FV1200, Olympus).

**Results**:

All positive control cells presented cytopathogenic effects after 3 days of incubation and positive labelling of N-protein whereas negative control cells remained intact. Additionally, all cells presenting cytopathogenic effects were positive for N-protein expression. One well was positive for N-Protein expression whereas no cytopathogenic effects was observed and was thus considered as positive for viral multiplication.

The first cytopathogenic effects on *ex vivo* samples were observed after 4 days of incubation. Five over 9 H24 central cornea media and 3 over 9 H24 corneoscleral rim media promoted viral multiplication in Vero-E6 cells. Among these 8 H24 positive samples, 4 of them were associated with a paired H0 medium promoting cytopathogenic effects, suggesting the presence of infecting viruses from the initial infection solution at H0 and a possible persistence of these viruses after 24h of incubation.

**Discussion** : The results of the infection of Vero-E6 cells with the organ culture media from the study 2 showed little evidence of production of infectious viral particles during the *ex vivo* infection. Indeed, half of the H24 media didn’t show any signs of viral multiplication. These results could be explained by the weak probability of cell infection when using a low viral load as described by **Jones et al., 2021; La Scola et al., 2020.** Moreover SARS-CoV-2 variable stability after 16-month-storage at -80°C and thawing could have impacted the number of viral particles used in this experiment. To note, the initial infection solution stored in the same conditions and further diluted to 1/100 was able to induce cytopathogenic effect after short-term incubation period (3 days) with Vero-E6 cells. This observation further supports the low or absent production of infectious viral particles in the different *ex vivo* organ culture media. These results could support either the possibility of an abortive process during the SARS-CoV-2 infection on ocular tissues or a very slow multiplication process (which would take more than 24h).

Taken together, these results suggest either a low and variable production of infectious viral particles or an abortive process in the central cornea and corneoscleral rim samples of the *ex vivo* study, allowing an increase in viral RNA but not formation of virions. In the absence of straightforward experimental clues in favor of the production of infectious viral particles during the *ex vivo* infection of human corneas, the conclusions of our experiment should be limited to the observation of viral genetic material replication. We thus have further discussed this limiting aspect of the study in the discussion (L607).

NEVERTHELESS, WE LEAVE IT TO THE DISCRETION OF THE REVIEWER AND EDITOR WHETHER OR NOT TO INCLUDE IN THE ARTICLE THIS ADDITIONAL EXPERIMENT WHICH STILL SUGGESTS A VERY LOW RISK OF VIRAL REPLICATION.

**References :**

Jones TC, Biele G, Mühlemann B, Veith T, Schneider J, Beheim-Schwarzbach J, Bleicker T, Tesch J, Schmidt ML, Sander LE, Kurth F, Menzel P, Schwarzer R, Zuchowski M, Hofmann J, Krumbholz A, Stein A, Edelmann A, Corman VM, Drosten C. Estimating infectiousness throughout SARS-CoV-2 infection course. Science. 2021 Jul 9;373(6551):eabi5273. doi: 10.1126/science.abi5273. Epub 2021 May 25. PMID: 34035154.

La Scola B, Le Bideau M, Andreani J, Hoang VT, Grimaldier C, Colson P, Gautret P, Raoult D. Viral RNA load as determined by cell culture as a management tool for discharge of SARS-CoV-2 patients from infectious disease wards. Eur J Clin Microbiol Infect Dis. 2020 Jun;39(6):1059-1061. doi: 10.1007/s10096-020-03913-9. Epub 2020 Apr 27. PMID: 32342252; PMCID: PMC7185831.

- Table 1: How do the authors explain the fact, that two of the three PCR positive cornea and culture medium were tested negative in Day2 and positive in Day10. In this case I would be very reluctant to speak of clear positive virus detection, especially considering the Ct value.

The experimental scheme for this part was apparently not clear enough and we updated it to ease its understanding (L378). Indeed, the two corneas (and respective culture medium) from one donor were analyzed randomly either at D2 or at D10. It was not a test-retest. Thus, we were able to detect viral RNA in one eye at a time point but not in the other eye at the other time point (unilateral contamination). This was previously reported by **Sawant et al., 2021.**

**References :**

Sawant OB, Singh S, Wright RE, 3rd, Jones KM, Titus MS, Dennis E, et al. Prevalence of SARS-CoV-2 in human post-mortem ocular tissues. Ocul Surf. 2021;19:322-9. Epub 2020/11/12.

- P 6 para 1: The authors scraped endothelial cells before infection with SARS-CoV-2 and maintained air contact of epithelial surface during culture. The authors explained the reasons for this setting ; however, this does not meet the usual corneal culture conditions in clinical routine. The authors should at least add a control group with intact endothelium layer and epithelium covered with medium.

As briefly explained in the methods section, we removed intentionally the endothelium as it represents an identified target tissue for SARS-CoV-2 entry and replication (**Zhou et al., 2020; Roehrich et al., 2020; Wassélius et al., 2003**), and thus a noteworthy bias source to explore the ocular surface infection in our experiment. The purpose of this experiment (study 2) was to mimic the infection that could occur at the ocular surface rather than mimic eye bank corneal storage conditions. Another aspect relates to the impact of endothelial removal on epithelial cells survival and/or function. The impact of endothelial removal has not been clearly established and the interdependence of the two tissues is still not clear. In this context, we chose a very short culture duration (24h) in order to limit any potential impact of endothelial removal on epithelial cell survival or on the interaction between viral particles and epithelial cells during incubation. In our experiment, we did not observe a significant difference in corneal thickness after removal of endothelial cells in human corneas in culture. We updated the text to improve the understanding and the scientific basis of this experimental aspect (L288):

“In order to eliminate potential replication in the endothelial cells, identified target cells for SARS-CoV-2 entry and replication [**16, 17, 29**] **Wassélius et al., 2003**), they were removed using a surgical microsponge and efficacy of scrapping controlled by Trypan blue staining (the Descemet membrane then turns completely blue).

**References:**

Zhou L, Xu Z, Castiglione GM, Soiberman US, Eberhart CG, Duh EJ. ACE2 and TMPRSS2 are expressed on the human ocular surface, suggesting susceptibility to SARS-CoV-2 infection. Ocul Surf. 2020;18(4):537-544. doi:10.1016/j.jtos.2020.06.007 PMID: 32544566

Roehrich H, Yuan C, Hou JH. Immunohistochemical Study of SARS-CoV-2 Viral Entry Factors in the Cornea and Ocular Surface. Cornea. 2020 Dec;39(12):1556-1562. doi: 10.1097/ICO.0000000000002509. PMID: 32826650.

Wassélius J, Wallin H, Abrahamson M, Ehinger B. Cathepsin B in the rat eye. Graefes Arch Clin Exp Ophthalmol. 2003 Nov;241(11):934-42. doi: 10.1007/s00417-003-0782-x. Epub 2003 Oct 28. PMID: 14586591.

Minor Points: All the following points have been addressed in the text.

- P 4, para 2: Change "Bayoud et al" (Ref 19) to "Bayyoud et al"
- P 12, para 3: The sentence beginning with "These results support the possibility ….." should be included in discussion, not in results section. The sentence was removed totally because this aspect is already addressed in the discussion.
- Even a non-native speaker recognizes linguistic deficiencies in the present manuscript. Please revise the manuscript with the help of a native speaker.
- Page 13: Table1: Were the samples taken from the periphery or the center of the cornea?

Contrary to the corneas used in the *ex vivo* infection model (study 2), the complete corneas (no processing/cutting of the tissue before RNA extraction) were used to evaluate the presence of viral genetic material at the different time points of standard organ culture. We clarified this aspect in the method section (L395) and added the volume of culture medium used for RNA extraction for each sample.

**Reviewer #3**: The work under review attempts to elucidate the risks of corneal transplantation as a vehicle for SARS-CoV-2 transmission. The work is generally well written and very timely, as fear of COVID-19 due to corneal transplantation has slowed use of an urgently needed restorative eye surgery across the globe. Although given the realities of the current pandemic, it is difficult to imagine that eye banks would not test for the presence of virus, whether it be in the donor's nasopharynx, the donor cornea, or both, the current study seeks to clarify the issues with experimental data so that a rational strategy for corneal procurement can be developed.

Some of the work is not novel. Others have assessed the presence of ACE2 and its co-receptors on the ocular surface and found similar results.

Indeed, as rightly pointed out, a few studies reported the presence of ACE2 and TMPRSS2 in human corneas, but, to the best of our knowledge, the expression of cathepsin B and L was not reported previously. In complement of these available data, our results propose a more detailed exploration of the expression pattern of these markers at the ocular surface. Our results also highlight an inter-individual variability in the expression of these markers, which is of clinical importance in the understanding of the SARS-CoV-2 ocular transmission risk.

The novelty of the paper is that the authors attempted to infect corneas ex vivo, which to this reviewer's knowledge, has not been done before. An additional strength of the study is the use of quantitative PCR

Comments:

- Page 3: regarding the macaque study, neither monkey developed conjunctival findings or showed any evidence for replication in the conjunctiva after topical ocular replication. Most likely the mild COVID-19-like respiratory disease seen in the two macaques was due to nasolacrimal drainage of virus.

Indeed, nasolacrimal drainage of virus after ocular topical instillation seems to be the more probable route of contamination in this experiment. Our references to this article have been updated accordingly in the introduction (L152-156):

“After ocular conjunctival inoculation (topically), they developed a moderate form of COVID-19 without any ocular symptoms and viral RNA was detected in conjunctival swabs 1 day after infection but not thereafter. Thus, a nasolacrimal drainage of the virus leading to the respiratory tract infection represents the most probable contamination pathway in this experiment[8]”

- Page 3: "whereas 12% of COVID-19 patients presented ocular symptoms" (ref 9). It is a shame to quote this reference in which 12% of critically ill COVID-19 cases had some eye finding, in most "congestion". There was no control group in this study. It is expected that a fair percentage of critically ill patients with other disorders would also have eye findings, most not directly related to their underlying disease.

We totally agree with the reviewer on this aspect and we thank him for pointing out the limitations of the article of Aggarwal et al., 2020. Indeed, the relevance and quality of the numerous studies dealing with this particular aspect was highly variable and the direct relationship between COVID-19 and ocular findings still discussed. We removed this reference and propose a modification of the text and references to better support the discrepancies in the literature reports (L156-162):

“Several studies reported that viral RNA was inconsistently detected in ocular surface samples (conjunctival swabs or tear samples) of confirmed COVID-19 cases (**Moreira et al., 2021**). Positivity rate in ocular surface samples of COVID-19 patients is likely dependent on the stage of the disease at the time of sampling and the sampling technique[10]. Likewise, highly variable prevalence of ocular lesions in patients affected by SARS-CoV-2 are also reported, ranging from 0.8 to 31.6%. (**Bal et al., 2021; Aiello et al., 2020**). Thus, the available data provide only limited and conflicting evidence to support a possible SARS-CoV-2 infection via the corneal or conjunctival route.”

**References:**

Moreira VM, Mascarenhas P, Machado V, Botelho J, Mendes JJ, Taveira N, Almeida MG. Diagnosis of SARS-Cov-2 Infection by RT-PCR Using Specimens Other Than Naso- and Oropharyngeal Swabs: A Systematic Review and Meta-Analysis. Diagnostics (Basel). 2021 Feb 21;11(2):363. doi: 10.3390/diagnostics11020363.

Bal S, Chodosh J, Venkateswaran N. Impact of SARS-CoV-2 on Ocular Surface Pathology and Treatment Practices: a Review [published online ahead of print, 2021 Aug 5]. *Curr Ophthalmol Rep*. 2021;1-6. doi:10.1007/s40135-021-00271-3

Aiello F, Gallo Afflitto G, Mancino R, Li JO, Cesareo M, Giannini C, Nucci C. Coronavirus disease 2019 (SARS-CoV-2) and colonization of ocular tissues and secretions: a systematic review. Eye (Lond). 2020 Jul;34(7):1206-1211. doi: 10.1038/s41433-020-0926-9. Epub 2020 May 18.

- Page 6: the authors might better explain the method of ex vivo infection, which seems nonstandard.

There is seemingly no standardized protocol for *ex vivo* infection of corneas by this virus. Thus, we were inspired by a previous work (**Alekseev et al., 2012**) with the following amendments:

- The removal of endothelial cells, which was performed, as explained above (p 26 of this document), to avoid any bias related to the potential artifactual multiplication of SARS-CoV-2 virus in endothelial cells.
- The use of a sterile non-woven compress: we have developed this technique to maintain preferentially the viral infectious solution on the corneal epithelium surface, in order to avoid an absorption of the infectious solution and thus entry of viral particles by the stroma. As the corneas were cut into pieces and large portions of stroma were exposed during the infection procedure. The aim of this approach is to mimic in the most similar way possible the contamination of conjunctival or corneal epithelial cells by contact with the virus as it may occur in humans.
- The use of pieces of corneal tissues, is based on two reasons:
  - the biological need to discriminate corneal and scleral + conjunctival tissue, that express differentially the SARS-CoV-2 receptors required for virus entry in the cells
  - the highly limited number of available samples to perform the *ex vivo* experiment.

**Reference:**

Alekseev O, Tran AH, Azizkhan-Clifford J. Ex vivo organotypic corneal model of acute epithelial herpes simplex virus type I infection. J Vis Exp. 2012 Nov 3;(69):e3631. doi: 10.3791/3631. PMID: 23149439; PMCID: PMC3514049.

- Page 7 " positive strand represented end products of viral." Please finish the sentence

We thank the reviewer for pointing out this incomplete sentence. We updated the text with the relevant information (L336-340):

“Negative RNA strands represent specifically the amount of replication matrix present in the cells, whereas positive strands represent the matrix used both for transcription (Sub genomic mRNA [SgmRNA]) and for formation of the virion genome. As the RdRp gene is located near the 5’ extremity of SARS-CoV-2 genomic RNA, the probability of SgmRNA amplification is very low. Based on this hypothesis, we approximated the increase in positive strand RNA with an increase in viral genomic RNA.”

- Page 12: it is unclear to this reviewer what the relevance is for totaling the positive and negative strand PCR products, to show significance, when productive replication of viral RNA would be indicated by the former and not the latter.

We thank the reviewer for this relevant question that supports a need for more detailed explanations in the method section (L330):

“Total viral RNA (IP4), positive (IP4+) and negative (IP4-) RNA strands were amplified in separated runs: Both primers were used for the detection of total viral RNA whereas only forward or reverse primers were added to the reaction mix for the detection of positive and negative RNA strands.”

Indeed, we performed 3 separate amplifications of the RdRp gene as explained. Incidentally, the IP4 total data do not represent the addition of positive and negative strand results. We performed these separated analyses to better confirm the work hypotheses:

- IP4 total and positive strands would show similar variation pattern, and should be increased in case of viral RNA multiplication
- Negative strands quantity would be moderately impacted by the viral RNA multiplication

- Page 14: I do not see the data supportive of the first sentence in the Discussion, that "SARS-CoV-2 replication was detected in fresh human cornea and conjunctiva" (or on page 14: "we documented for the first time ...". That would require evidence of production of infectious viral particles in these tissues, whether by increasing culture titers, immunohistochemistry for intact viral particles, or electron microscopic evidence of intact viral particles.

Indeed this pertinent remark points out the overinterpretation of the results in this sentence. We modified it accordingly (L563), as we observed an increase in total and positive viral RNA but we did not get experimental clues to support virion formation by both immunohistochemistry, electron microscopy.

We additionally evaluated the production of infectious viral particles by incubating the *ex vivo* infection media sampled at H0 and H24 on Vero-E6 cells and searching for ECP (see details in p20 of the present document). The results of this experiment do not allow us to conclude definitively on the presence of infectious viral particles in the tested samples. We thus have further discussed this limiting aspect of the study in the discussion (L606).

NEVERTHELESS, WE LEAVE IT TO THE DISCRETION OF THE REVIEWER AND EDITOR WHETHER OR NOT TO INCLUDE IN THE ARTICLE THIS ADDITIONAL EXPERIMENT WHICH STILL SUGGESTS A VERY LOW RISK OF VIRAL REPLICATION.

- Page 15: again regarding the macaque study, the authors of that study found viral RNA at 1 day post conjunctival inoculation, not virus, and it was gone thereafter. This would not be consistent with viral replication in the conjunctiva:

As discussed above concerning this article, we clarified the information that relates to the findings of this study and removed non-relevant citations.

Page 15: regarding "SARS-CoV-2 replication after 24h was moderate, with only total viral RNA showing a significant increase", an increase in total viral RNA without increased positive strand RNA is not equivalent to replication of the genome, and may reflect only an **abortive process.**

This remark points out again different aspects relative to RNA replication results and methodology used in our study. As detailed above, we indeed have hypothesized that the positive strand amplified using RdRp gene can be approximated to genomic RNA due to its close proximity to the 5' end and the low risk of subgenomic amplification. Moreover, due to the modification of statistical analysis (for non-normally distributed data), the increase in positive strand is significant and parallels the significant increase observed for total RNA.

In response to your and other reviewer requests, we carried out additional tests (described p23 of the present document). We didn’t formally demonstrated the production of infectious viral particles despite an initial infection solution highly concentrated in viruses (10^7 TCID50/mL) which could not be compared to the conditions of a natural contamination via aerosols/droplets.

We thus have further discussed this limiting aspect of the study in the discussion (L607).

Page 16: " were associated with effective and moderate replication of the virus after experimental infection of freshly excised human corneas". The data supplied does not support this statement.

Indeed, the reviewer highlights an over-interpretation of the results in this sentence. We changed the text accordingly to support that we only evidenced RNA multiplication and not a production of new infectious virions (L608-610).

This reviewer agrees however with the final sentence and conclusion of the paper.
