# Supplementary material for: Exploration of the ocular surface infection by SARS-CoV-2 and implications for corneal donation: An ex vivo study
Source: PLoS Med. 2022 Mar 1;19(3):e1003922. doi: 10.1371/journal.pmed.1003922 (PMC8887728; doi:10.1371/journal.pmed.1003922)
Supplement: Attachment — Submitted filename: R2_Response to editors.docx [file pmed.1003922.s017.docx]

**COVER LETTER**

Saint-Etienne, January 17^th^, 2022

Dear Editor,

Please find attached a revised version of our article titled “**Exploration of the ocular surface infection by SARS-CoV-2 and implications for corneal donation.** **An *ex vivo* study**” (PMEDICINE-D-21-02421R2)

We have carefully responded to all the points addressed by the editors.

As per your instructions, all amendments in the revised version are stated in our point-by-point response and are marked in red in the article.

We greatly hope that this new version will meet the editors' expectations.

Yours sincerely,

Professor Gilles THURET, MD, PhD

Saint-Etienne University Hospital

"Corneal Graft Biology, Engineering, and Imaging" Laboratory EA 2521, SFR143

Faculty of Medicine

Saint-Etienne

France

Institut Universitaire de France, Paris

Requests from Editors:

1. Response to reviewers: As suggested by Reviewer 2, please do include the additional experiment to assess whether infected tissue (Study 2) contained replicable SARS-CoV-2 virus.

Accordingly, we have added this additional experiment in methods (L342) and in results sections (L484). We also updated the discussion section with respect to the results of this experiment (L572).

2. Title: Please capitalize the first word of the subtitle, and please update the title in the manuscript submission system accordingly: “Exploration of ocular surface infection by SARS-CoV-2 and implications for corneal donation: An ex vivo study.”

The requested modification was performed accordingly.

3. Page 1-2: Please remove the sections titled “Grant” and “Author contributions” from the main text. Please ensure this information is completely and accurately entered into the manuscript submission system.

These sections were removed from the manuscript and the related information checked in the submission system.

4. Abstract: Lines 55-58: We suggest removing the numbering from the objectives: “The aims of
this study are to specifically assess the capacity of human cornea to be infected by SARS-CoV-2 and promote its replication ex vivo, and to evaluate the real-life risk of corneal contamination by detecting SARS-CoV-2 RNA in corneas retrieved in donors diagnosed with COVID-19 and non-affected donors.”

5. Abstract: Methods and Findings: Please remove the numbering from the description of the methods. At line 60 we suggest: “To assess the capacity of human cornea to be infected by SARS-CoV-2, the expression pattern of SARS-CoV-2 receptor ACE-2 and activators TMPRSS2 and Cathepsins B and L in ocular surface tissues from non-affected donors was explored…” Please revise similarly for parts 2 and 3.

6. Abstract: Line 64: Please define “TCID” in the text.

7. Abstract: Line 71-72: Please note that the spatial patterns of expression were the derived from the immunohistochemistry experiments.

8. Abstract: Line 73: Please clarify “IP4 strands” for non-specialists.

9. Abstract: Line 73-77: Please clarify if the comparison is between 24 hr and 30 minute time points.

10. Abstract: Line 84: Please remove “a significant” from the sentence.

Accordingly, the abstract section has been updated following the previous suggestions (5 to 10).

11. Author summary: Please format the author summary into 3 sections each with 3-4 bulleted points. For example, we suggest:

Why Was This Study Done?

-Corneal transplantation is by far the most common transplantation procedure in the world and there is a severe lack of corneal donation to respond to the increasing patient need.

-The persistent COVID-19 pandemic has dramatically worsened this situation by limiting the access to corneas during a long period of the pandemic and finally increasing the long list of contraindications to corneal donation based solely on an exacerbated precautionary principle.

-Exploration of the SARS-CoV-2 risk of transmission through corneal transplantation is mandatory to adapt the donor selection strategy and improve corneal tissue procurement in the persisting COVID-19 crisis.

What Did the Researchers Do and Find?

-We explored and observed the expression of 4 proteins related to molecular pathways allowing SARS-CoV-2 cellular entry at the ocular surface (cornea, bulbar conjunctiva, 10 samples). These proteins were differentially located in the studied tissues and their expression was variable among donors.

-The experimental infection of five freshly excised corneas with highly concentrated SARS-CoV-2 viral solution promoted only moderate and variable viral RNA multiplication in corneal samples. Twenty-four hours after inoculation, a significant increase in total and positive strand viral RNA was observed in the epithelium of central cornea (p-value: 0.0039 for and 0.0117 respectively) and in corneoscleral rim (p-value: 0.0039 and 0.0391 respectively).

-We explored the presence of SARS-CoV-2 viral genetic material in corneas and respective storage media from donors tested positive or negative to COVID-19 PCR on nasopharyngeal swab. All samples from the 159 donors not contaminated by COVID-19 were negative 111 for SARS-CoV-2 RNA. SARS-CoV-2 RNA was detected in only 3 corneas out of 28 corneas (11%) from donors diagnosed with COVID-19.

What Do These Findings Mean?

-Our results support that the ocular surface expresses in a variable way the proteins involved in SARS-CoV-2 cellular entry and infection. We observed a moderate and variable increase in total and positive viral RNA after experimental infection of human freshly excised corneas. A very low rate of positivity in donors diagnosed with COVID-19 was observed, supporting a low risk of SARS-CoV-2 presence in donor corneas.

-This work suggests that it would be beneficial to procure corneas from donors tested positive to COVID-19 (nasopharyngeal sawbs), test their corneal storage media and transplant the negative corneas.

The author summary has been updated accordingly (from L65 to L96).

12. Introduction: Line 135-136: We suggest revising to: “Finally, it deprives some patients and/or families of access to tissue donation, which can be a beneficial aspect of the mourning process…” or similar, depending on your meaning.

We have revised this sentence following the editors’ suggestion (L112)

13. Methods: Line 249: Please clarify (H0) in the text.

H0 meaning was defined on L226.

14. Methods: Line 284: Please define the abbreviation “TCID” in the text.

TCID abbreviation was defined accordingly on L261.

15. Methods: Line 323: We suggest including a reference for the online PCR protocol, rather than inserting the link into the text.

Accordingly, the reference to this protocol was added as the reference number 31 (L301), and reference numbering updated accordingly.

16. Methods: Line 388-389: PLOS does not permit "data not shown.” Please remove this claim, or do one of the following: If you are the owner of the data relevant to this claim, please provide the data. If the data not shown refer to a study from another group that has not been published, please cite personal communication in your manuscript text (it should not be included in the reference section). Please provide the name of the individual, the affiliation, and date of communication. The individual must provide PLOS Medicine written permission to be named for this purpose.

To clarify this point, we included the relevant data and methods in the supporting information (S1 file, L133).

17. Methods: Line 410-412: For each result presented (e.g. in the respective table legend) please note the test that was used.

Accordingly, we have added the information about the performed statistical test in the legend of figure 4 (L478) and figure S5 (L830 and S1 file)

18. Discussion: Line 533: Please revise to “may not be a good viral vector” or similar.

19. Discussion: Line 636: We suggest tempering this slightly “Our results suggest that only corneas with positive PCR…”

20. References: For reference 4 and 5, if these are online materials, please provide the weblink, and please check that formatting is correct: Please use the "Vancouver" style for reference formatting, and see our website for other reference guidelines <https://journals.plos.org/plosmedicine/s/submission-guidelines#loc-references>

Please check formatting of reference 11.

Reference formatting has been updated according to editors’ suggestion and following reference guidelines.

21. Figure 1: Please define “OC” in the legend.

Figure 1 has been updated accordingly. OC was replaced by “culture medium”.

22. STROBE Checklist: Thank you for including the checklist. Please include the checklist as a separate file (e.g. S1 Checklist).

Please revise the checklist to remove any references to page numbers. Please refer only to sections and paragraph numbers.

STROBE checklist has been checked accordingly and was included in a separate file names S1 checklist.

Comments from Reviewers:

Reviewer #2: The authors addressed all major points and performed additional experiments as requested. The manuscript improved substantially. Notably, clear evidence of replicable virus in corneas in study 2 could not be shown by the authors despite the well performed additional experiments. However, the authors improved the discussion accordingly and pointed out the possibility of an abortive process. Taken together, the data supports the conclusions and the demand to question current donor selection algorithms in view of the corneal donor shortage. I would suggest including the additional experiment in the paper, in my opinion it gives relevant additional information and can contribute to reduce "positive publication bias". Taken together, the paper represents an important contribution to the present debate on donor selection and eye bank procedures in the COVID 19 pandemic, I recommend to publish now without further changes.
